# A rare case of three years disease free survival in a locally advanced parathyroid carcinoma successfully excised by complete surgical resection

**DOI:** 10.1016/j.amsu.2020.07.023

**Published:** 2020-07-18

**Authors:** Abdul Ahad Sohail, Bushra Ayub, Syed Akbar Abbas, Shafqat Ali Sheikh, Talha Ahmed Qureshi, Muhammad Usman, Asad Diwan

**Affiliations:** a Department of Surgery, Aga Khan University Hospital, Pakistan; b Patel Hospital, Pakistan; c Aga Khan University Hospital, Pakistan; d Department of Histopathology, Aga Khan University Hospital, Pakistan

**Keywords:** Parathyroid carcinomas, Primary hyperparathyroidism, Complete surgical resection, Thyroid lobectomy

## Abstract

Parathyroid carcinoma (PC) is one of the rarest malignancies making approximately 0.005% of all cancers. It may arise sporadically or less commonly, in conjunction with genetic endocrine syndromes. Due to the rarity of the disease, no general consensus or definitive guidelines exist for its pre-operative diagnosis, management, or follow up. Surgical tumor removal is the gold standard treatment to prevent its recurrence. Parathyroid carcinoma has a high recurrence rate ranging from 40 to 60% in recent literature. We report a case of a seventy-year-old elderly female with locally advanced parathyroid carcinoma successfully surgically excised completely with a 3 year disease free survival period without adjuvant chemotherapy or radiotherapy.

## Highlights

• Incidence of Parathyroid carcinoma is 0.005%.
• Presented as Thyroid Adenoma.
• No other clinical signs of systemic disease.
• Excised completely with surgical resection.

## 1 Introduction

Parathyroid carcinoma (PC) is one of the rarest malignancies making approximately 0.005% of all cancers [1,2]. Recent literature suggests that it accounts for only 0.5–5% of all the cases of primary hyperparathyroidism, with less than 1000 cases reported in the literature since it was first discovered in 1904 [[3], [4], [5]]. It may arise sporadically or less commonly, in conjunction with genetic endocrine syndromes such as multiple endocrine neoplasia (MEN) type 1, type 2A, and hyperparathyroidism jaw-tumor syndrome (HPT-JT) [[6], [7], [8]]. Diagnosis is usually difficult and challenging due to the absence of clinical and radiological characteristics that reliably distinguish benign from malignant disease [1,2,6]. Due to the rarity of the disease, no general consensus or definitive guidelines exist for its diagnosis, management, or follow up [6,8,9].

According to recent literature, 90% of parathyroid carcinoma cases are hormonally functional on presentation with clinically apparent symptoms of hypercalcemia and severe primary hyperparathyroidism which include anxiety, depression, loss of appetite, and weight loss usually present with the renal and skeletal disease [4,6,10]. Serum calcium is often elevated with greater than 14mg/dl and serum parathyroid hormone is often raised to about three to ten times the upper limit of normal [1]. Surgery is the mainstay of treatment with complete resection of the tumor to achieve microscopically negative margins to prevent recurrence [1,2,6,7,11].

We report a case of an elderly female who presented to us with initial suspicion of parathyroid adenoma. However, post-operatively she was diagnosed with parathyroid carcinoma on histopathological examination.

## 2 Case report

**Fig. 1:**
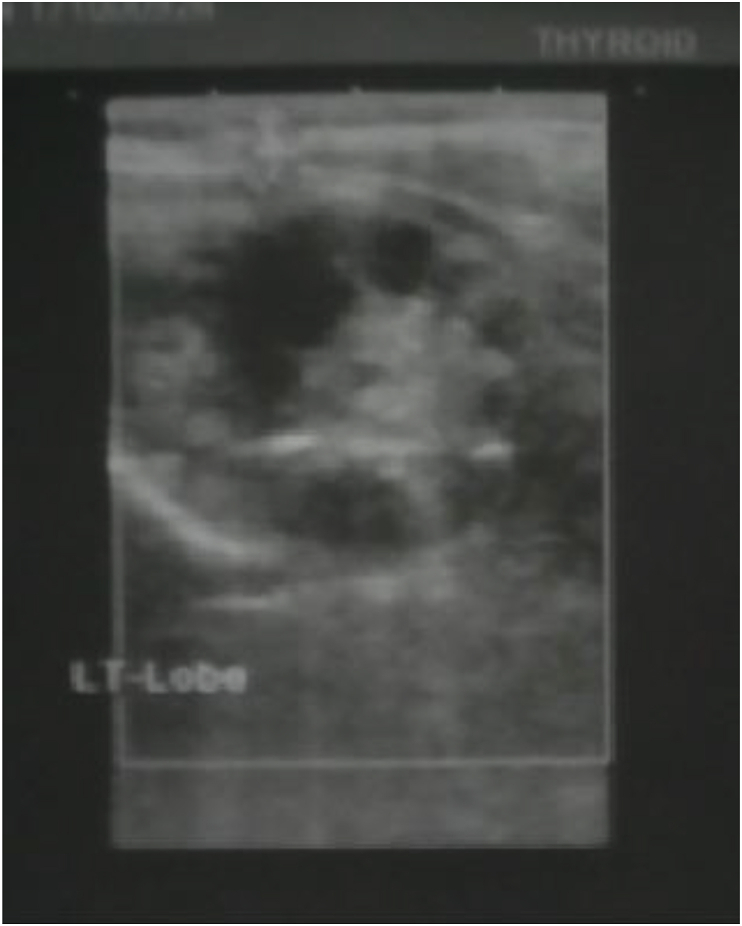
A 3.6 × 2.8cm complex solid and cystic nodule is seen on left side close to left lower margin of thyroid measuring.

This is a case of 70 years old female, with no known co-morbidities, who presented to the Otolaryngology clinic with complaints of increased somnolence and reduced appetite for past the 3–4 years. These complaints were not associated with any other associated symptoms. Her past medical history and family history were unremarkable. She had a history of the cesarean section under spinal anesthesia 30 years ago. On examination, she was well oriented to time, place, and person. She had a heart rate of 64 beats per min, a respiratory rate of 16 breaths per min, a blood pressure of 110/70 mm Hg, and she was afebrile. Her general physical and systemic examination was also unremarkable. Her serum calcium was 14.1mg/dl (8.8–10.6) and serum parathyroid hormone showed a value of 457mg/dl (7-53). Her vitamin D level, serum albumin, and creatinine were all within a normal range. 24-hour urinary calcium and phosphorus were also within normal limits. She underwent neck ultrasonography which showed a complex solid and a cystic nodule on the left side close to the lower pole of the thyroid measuring 3.6 × 2.8 cm (Fig. 1).

**Fig. 2:**
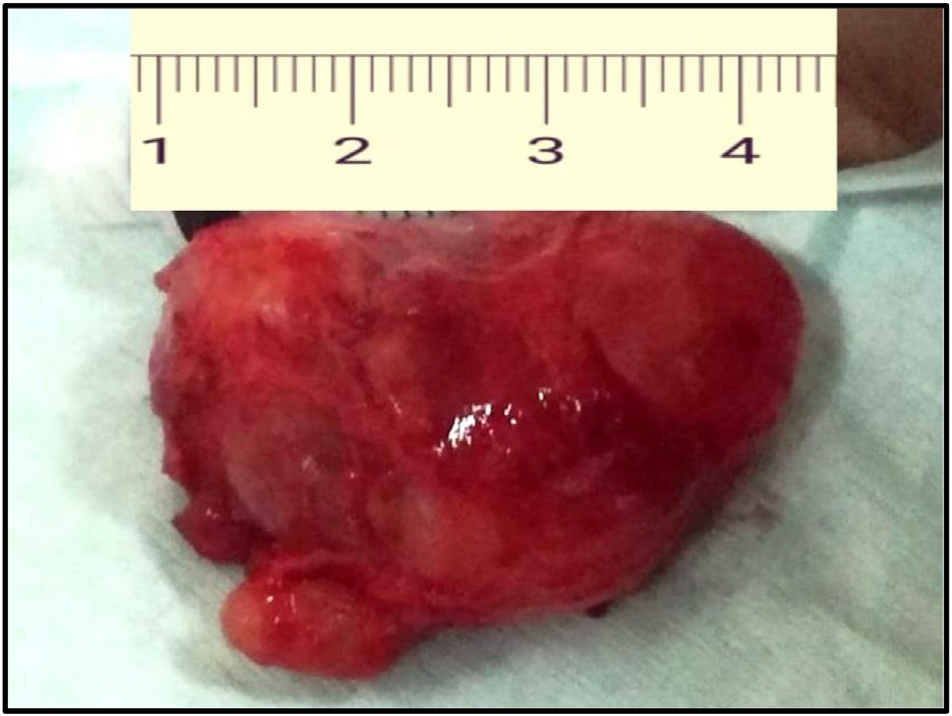
Parathyroid carcinoma along with thyroid lobe after resection.

The clinical picture was suggestive of a parathyroid adenoma and she was admitted electively for its resection. After relevant investigations and anesthesia fitness, she underwent complete excision of the left parathyroid lesion and left lobectomy of thyroid gland under general anesthesia. Intra-operative findings were enlarged left parathyroid gland which was inseparable from the multi-nodular left lobe of the thyroid gland. A recurrent laryngeal nerve was identified and saved. Neck drain was placed and the sample was sent for histopathology (Fig. 2). The lesion was not involving any other structure other than the thyroid gland. No level VI lymphadenopathy was observed intraoperatively.

The patient remained hemodynamically stable with no signs of recurrent laryngeal nerve palsy. Her post-op serial serum calcium monitoring was done which decreased to normal range and remained normal. No other signs of hypocalcemia were noted and serum calcium was within a normal range. The patient remained stable and hence was discharged as post-op day 2 on oral calcium supplements and analgesia.

**Fig. 3:**
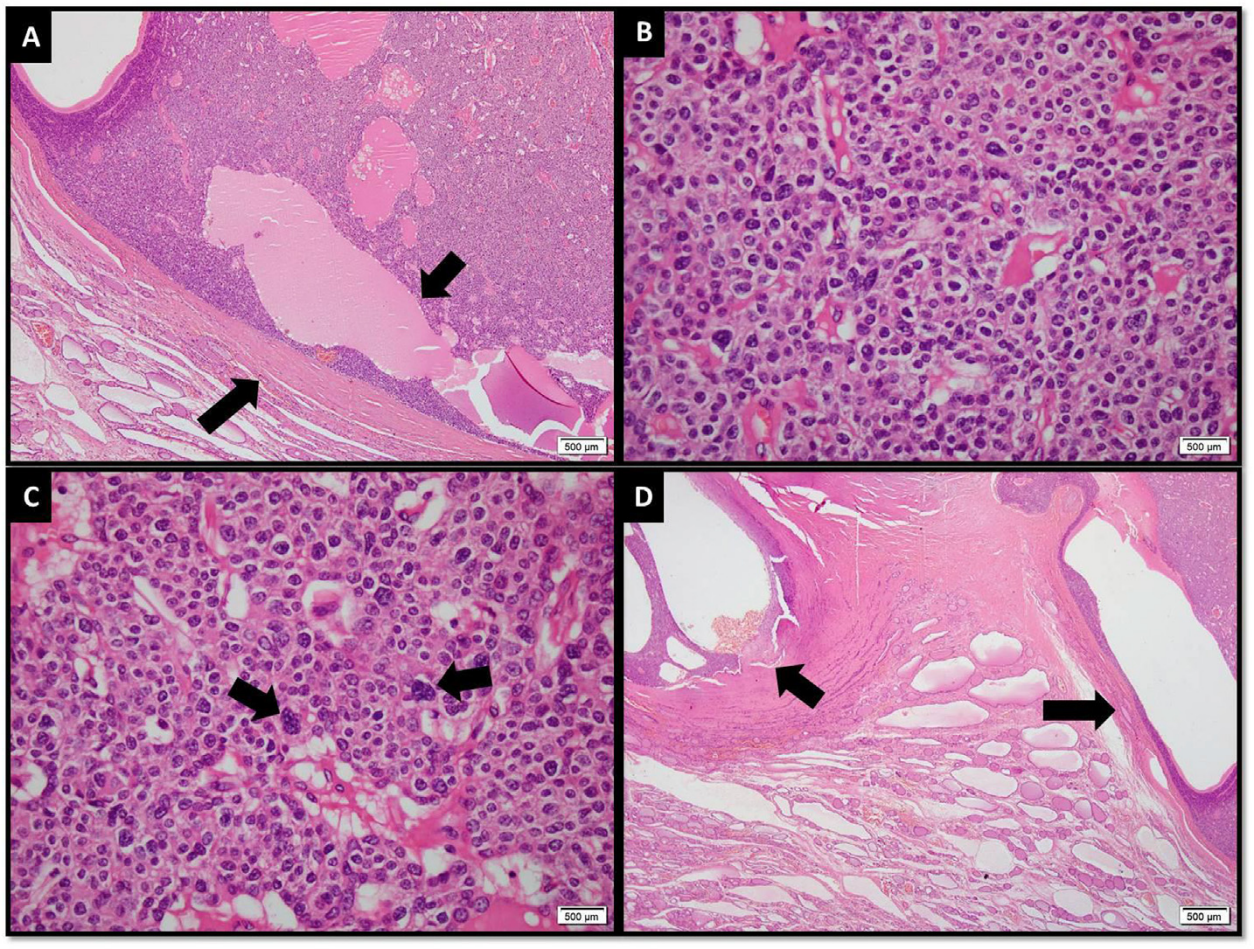
[A] Low power view of the neoplastic lesion exhibiting fibrous capsule (longer arrow), sheets of cells and cystic spaces (shorter arrow) [B] The neoplastic cells have pale to eosinopilic cytoplasm and vesicular nuclei with inconspicuous nucleoli [C] Few cells showed marked nuclear pleomorphism (arrows) [D] Focal invasion of parathyroid capsule (longer arrow) and extension into thyroid tissue is also seen (shorter arrow).

At 1-week follow-up her serum calcium was 8.9mg/dl and serum parathyroid hormone was 16.3pg/ml. Histopathology revealed a neoplastic lesion composed of closely space clusters of medium-sized ovoid to polygonal cells with pale to mildly eosinophilic cytoplasm, distinct cell borders, and centrally placed round to ovoid nuclei showing prominent nucleoli. Moderate cytological atypia and nuclear pleomorphism were seen. Occasional mitotic figures were consistent with parathyroid carcinoma. The tumor was seen invading into the thyroid parenchyma 0.4cm away from the resection/capsular margin (Fig. 3).Her repeat serum calcium was also within the normal range. MEN syndrome workup was done which was negative. The patient was seen regularly for 1 year and then has been on regular follow up yearly for past three years. She has been doing well with no symptoms and signs for recurrence of disease. Hence she has an overall disease free survival of three years with only complete surgical resection of tumor and no chemotherapy or radiotherapy.

## 3 Discussion

Parathyroid carcinoma (PC) is one of the rarest endocrine malignancies with an incidence of 0.015 per 100,000 population in the United States [1,12]. These tumors are hormonally functional with less than 38 cases reported in the literature for non-functional parathyroid carcinoma [6]. Hence patients often present with signs and symptoms of hypercalcemia hyperparathyroidism [6]. Multiple case series suggest the involvement of renal and skeletal disease in up to 50% of patients in parathyroid carcinoma with an incidence of nephrolithiasis and renal insufficiency as high as 56 and 84% respectively [13]. Recent studies also suggest the presence of a palpable cervical mass in about 30–76% of patients while the incidence of completely asymptomatic parathyroid carcinoma described in 7–46% of patients and approximately 25% of patients develop distant metastases at some point during the disease [6,18]. In contrast, our patient had a hormonally functioning parathyroid carcinoma but she did not develop renal or skeletal disease despite advanced age and did not have a palpable neck mass.

The pre-operative diagnosis of parathyroid carcinoma is difficult and challenging due to the absence of diagnostic criteria and a lack of consensus in literature [8,9]. It should be suspected in a patient with a very high serum calcium level of greater than 14mg/dl or an increased parathyroid hormone of greater than three to ten times of upper limit of normal palpable cervical lymph nodes should also raise suspicion of PC [9,14]. Neck ultrasonography and parathyroid scintigraphy have been described as the imaging of choice for detecting parathyroid lesions and a combination of both raises diagnostic accuracy and sensitivity; however, they cannot reliably distinguish benign from malignant disease [6,8,11]. A size of greater than 3cm on ultrasonography should raise a suspicion of PC [15]. Fine needle aspiration cytology should be avoided in lesions that are suspected to be PC as it increases the risk of needle tract dissemination, parathyromatosis, and recurrence [6]. Hence the post-operative diagnosis of parathyroid carcinoma on histopathological examination appears to be a norm rather than the exception [6].

The gold standard for the treatment of parathyroid carcinoma is complete surgical excision of the tumor achieving microscopically negative margins. There is a limited role of chemotherapeutic agents and radiotherapy in uncomplicated cases [6,7,9,16]. There is a lack of evidence on prophylactic neck dissection. The incidence of neck metastasis has been reported to be 1–6% [6].

There is a high rate of locoregional recurrence rate ranging from 40 to 60% with nearly half of patients developing distant metastasis [7,17]. Recurrence of the tumor is common within 2–3 years of initial surgery. A close follow up and tumor surveillance is required. Serial neck ultrasonography, serum calcium, phosphate, and parathyroid levels are required [6] 5- year disease-free survival rate after complete surgical removal ranges from 76% to 85% [6]. This data is similar to our case described here as our patient has a disease free survival of three years after only complete surgical excision of tumor without adjuvant chemotherapy or radiotherapy and is still on regular follow ups.

## 4 Conclusion

The case of parathyroid carcinoma described here presents two unique features. Parathyroid cancer may present as a parathyroid adenoma and hence complete surgical excision of suspicious malignant lesion intra-operatively and close follow up post-operatively can provide an excellent long term disease free survival rate in patient without adjuvant chemotherapy or radiotherapy. Secondly, complications associated with hyperparathyroidism may be absent in parathyroid carcinoma. Therefore clinicians should have a high index of suspicion for parathyroid malignant lesions if the imaging shows unusual characteristics. Thyroid lobectomy should be performed to enhance the clearance of malignant disease whenever deemed necessary.

## Funding sources

None.

## Consent

Written informed consent was obtained from the patient for publication of this case report and accompanying images. A copy of the written consent is available for review by the Editor-in-Chief of this journal on request.

## Declaration

The above case has been reported in line with SCARE 2018 criteria [19].

## Provenance and peer review

Not commissioned, externally peer reviewed.

## Ethical approval

This case report is exempted from ethical review of our institute.

## Author contribution

Abdul Ahad Sohail. Wrote the initial draft of case report. Collected all relevant data from files and patient. Finalized the case report. Literature Search. Bushra Ayub: Wrote the initial draft of case report. Collected all relevant data from files and patient. Finalized the case report. Literature Search. Syed Akbar Abbas: Was involved in critical review of the case report, further editing and finalizing of the case report. Shafqat Ali Sheikh: Was involved in critical review of the case report, further editing and finalizing of the case report. Talha Ahmed Qureshi: Was involved in critical review of the case report, further editing and finalizing of the case report. Muhammad Usman: Was involved in arranging figures in case report and providing histopathology pictures. Also finalized the case report. Asad Diwan: Was involved in arranging figures in case report and providing histopathology pictures. Also finalized the case report.

## Registration of research studies

1. Name of the registry:

2. Unique identifying number or registration ID:

3. Hyperlink to your specific registration (must be publicly accessible and will be checked):

## Guarantor

Abdul Ahad Sohail.

Syed Akber Abbas.

## Declaration of competing interest

None.
